# Comparison of hepatic responses to glucose perturbation between healthy and obese mice based on the edge type of network structures

**DOI:** 10.1038/s41598-023-31547-2

**Published:** 2023-03-23

**Authors:** Yuki Ito, Shinsuke Uda, Toshiya Kokaji, Akiyoshi Hirayama, Tomoyoshi Soga, Yutaka Suzuki, Shinya Kuroda, Hiroyuki Kubota

**Affiliations:** 1 Division of Integrated Omics, Medical Research Center for High Depth Omics, Medical Institute of Bioregulation, Kyushu University, 3-1-1 Maidashi, Higashi-ku, Fukuoka, 812-8582 Japan; 2 Department of Computational Biology and Medical Sciences, Graduate School of Frontier Sciences, University of Tokyo, 5-1-5 Kashiwanoha, Kashiwa, Chiba 277-8562 Japan; 3 Data Science Center, Nara Institute of Science and Technology, 8916-5, Takayamacho, Ikoma, Nara 630-0192 Japan; 4 Institute for Advanced Biosciences, Keio University, 246-2 Mizukami, Kakuganji, Tsuruoka, Yamagata 997-0052 Japan; 5 Department of Biological Sciences, Graduate School of Science, University of Tokyo, 7-3-1 Hongo, Bunkyo-ku, Tokyo, 113-0033 Japan; 6 Core Research for Evolutional Science and Technology (CREST), Japan Science and Technology Agency, Bunkyo-ku, Tokyo, 113-0033 Japan

**Keywords:** Cell biology, Computational biology and bioinformatics, Diseases

## Abstract

Interactions between various molecular species in biological phenomena give rise to numerous networks. The investigation of these networks, including their statistical and biochemical interactions, supports a deeper understanding of biological phenomena. The clustering of nodes associated with molecular species and enrichment analysis is frequently applied to examine the biological significance of such network structures. However, these methods focus on delineating the function of a node. As such, in-depth investigations of the edges, which are the connections between the nodes, are rarely explored. In the current study, we aimed to investigate the functions of the edges rather than the nodes. To accomplish this, for each network, we categorized the edges and defined the edge type based on their biological annotations. Subsequently, we used the edge type to compare the network structures of the metabolome and transcriptome in the livers of healthy (wild-type) and obese (*ob*/*ob*) mice following oral glucose administration (OGTT). The findings demonstrate that the edge type can facilitate the characterization of the state of a network structure, thereby reducing the information available through datasets containing the OGTT response in the metabolome and transcriptome.

## Introduction

Biological phenomena include the interactions between various biochemical molecular species to form network structures. Therefore, an in-depth investigation of the network structures corresponding to statistical interactions, including biochemical interactions, will permit better understanding of biological phenomena^1,2^. In particular, when the biochemical reaction network is unknown, the network formed by statistical interactions that have been inferred from a dataset provides substantial information toward an understanding of these biological phenomena. Moreover, the clustering of nodes corresponding to molecular species and enrichment analysis are frequently used to determine the biological significance of network structures^2–4^. However, as these methods focus on the function of the nodes, the role of the edges, or the connections between nodes, has rarely been studied.

We consider that it is equally important to investigate the functions performed by closely connected nodes and the type of functional connections present within network structures. Moreover, additional information regarding the functions of the edges, such as the composition of the category of each edge as an indicator of the biological characteristics of the network, is helpful for examining the characteristics of the network structure.

To determine the nature of the functional connection, it is also necessary to perform functional annotation of the edges in the network. A pioneering study of “edge ontology,” to functionally annotate network edges, was reported by Lu et al. in^5^. Several classical pathway databases have been established over the past decade to document the published information on established pathways^6,7^. However, two problems arise when these classical pathways are documented. First, each database contains unique components of the same pathway. Second, as each pathway is visualized independently, the crosstalk between pathways remains unknown. Alternately, modern high-throughput techniques, such as large-scale yeast two-hybrid screens^8–12^ and large-scale databases, have allowed the construction of standard large-scale networks documenting protein–protein interactions; this development has ushered in a different perspective on pathway studies, including quantitative insights from the network structure analysis^1,2^.

Lu et al.^5^ attempted to embed an established classical pathway within a large-scale network; they embedded classical pathways in large-scale networks by superimposing classical pathways, including their edges, to preserve the informative edges associated with classical pathways. They attempted to elaborate on the process of network integration in this manner. However, as the same edge symbols had different meanings, the precise definition of the edge for the overlay was ambiguous. Thus, to conduct large-scale pathway mining, there is a critical need to develop accurate edge ontology that can represent the various types of relationships between pathway components. Lu et al. represented various edge types consistently and developed an edge ontology for their classification. Consequently, their proposed edge ontology enabled them to delineate four distinct types of pathways, as well as provide additional information demonstrating the significance of the edge ontology.

From the findings of Lu et al., we hypothesized that the network structure could be characterized by quantifying the distribution of the edge ontology, which could serve as a novel index for comparing network structures. For this purpose, we defined the edge ontology of the metabolites and genes to analyze the network structures. However, the use of edge ontology analysis has not been reported for multiple layers and metabolites. Additionally, the edge ontology analysis defined in this study differs slightly from conventional edge ontology. Consequently, to avoid confusion, it is hereafter referred to as “edge type (ET).”

The network structure is an integral part of ET-based network analysis. Generally, it is difficult for an “omics” measurement dataset to construct an entire network structure from biological knowledge. Therefore, it was critical to infer the network structure from the omics dataset before the ET analysis on the network was performed. In contrast to other methods using ordinary differential equations^13–15^ or regression to infer the network structure^16^, we used mutual information^17^.

Blood glucose levels are strictly maintained and impaired glucose tolerance leads to various diseases^18,19^. The liver plays an essential role in maintaining blood glucose levels, and part of the control of the metabolic response depends on the regulation of hepatic gene expression^19–21^. A recent study examined hepatic glucose metabolism using multiomics measurements^22,23^. However, the experimental design was inadequate for the examination of statistical independence. We retrieved metabolome and transcriptome data, previously acquired by our group, from 11 or 12 healthy mice (WT) prior to and following an oral glucose tolerance test (OGTT), which is a standard test used to diagnose diabetes and in which glucose administration was performed to determine if the patient has impaired glucose tolerance^24^. In this study, to gain a better understanding of the comprehensive hepatic responses to glucose perturbation, we examined the network difference between prior to and following the OGTT using ET. Additionally, we used obese (*ob*/*ob*) mice, which are leptin-deficient mutants with abnormal glucose metabolism^25,26^. Our comparison of the network responses of the OGTT in *ob*/*ob* mice with those in healthy mice based on the ETs was intended to reveal the anomalous response to glucose perturbation in obese mice. We first inferred the network structure using mutual information in WT and *ob*/*ob* mice and determined the difference network between prior to and following the OGTT, which we considered as the OGTT response network. The OGTT response networks were compared between WT and *ob*/*ob* mice and characterized abnormal glucose metabolism by the ETs. In addition, the distribution of ET was obtained by counting the ET that appears in the network. Thus, we propose that the distribution of ET can serve as a novel index to characterize the state of the network for each condition in a reducible manner and we demonstrate the utility of this index through a comparison of the network responses to the OGTT in *ob*/*ob* mice with those in healthy mice. Because the distribution of ET is almost unaffected by the local structure of the network, a robust characterization and comparison of network states can be achieved.

## Results

### Overview of our network analysis based on edge type

**Figure 1:**
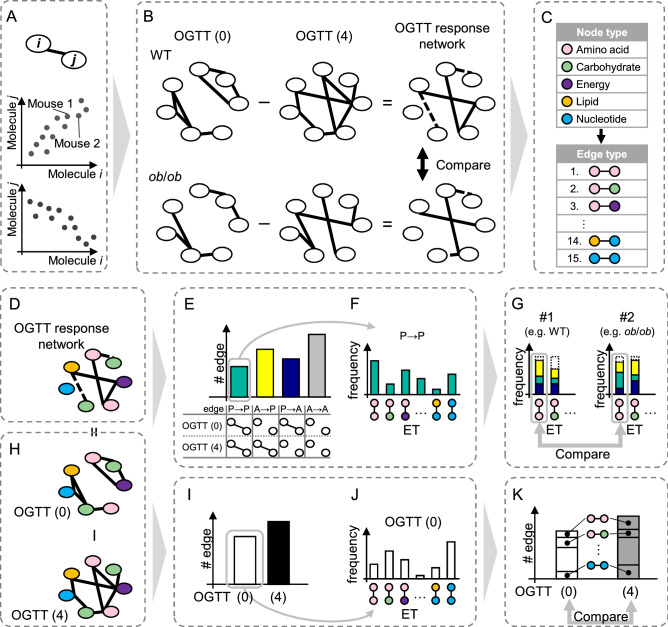
Schematic representation of the network inference, the network comparison, the definition of the edge type, and the comparison of ET distribution. (**A**) Evaluation of the relationships between the molecular species (metabolite or gene) by statistical independence. The nodes are connected by the edges, given that the mutual information^17^ between nodes is not statistically 0 (*p* < 0.05, permutation test). (**B**) Inference of the OGTT (0) network, the OGTT (4) network, and the OGTT response network and the comparison of the OGTT response network between WT and *ob*/*ob*. OGTT (0) and OGTT (4) represent for the conditions prior to and following the OGTT, respectively. (**C**) Definition of ETs. (**D**–**G**) Comparison of the OGTT response networks using the defined ETs. (**H**–**K**) Comparison of each condition’s networks using the ET distribution. (**D**, **H**) The OGTT response network and the network of OGTT (0) and OGTT (4). Nodes with different colors are different node types. (**E**) The number of the OGTT response edges. “P → P,” “A → P,” “P → A,” and “A → A” stand for the edges that were are common to the OGTT (0) and OGTT (4) networks, the edges only existing in the OGTT (4) network, the edges only existing in the OGTT (0) network and the edges existing in neither OGTT (0) nor OGTT (4) networks, respectively. (**F**) The ET distribution of each OGTT response edge. An example for P → P edges is shown. (**G**) The comparison of OGTT response edge distribution between each OGTT response network. #1 and #2 stand for the OGTT response network 1 and 2, respectively. (**I**) The number of each condition’s edges. (**J**) The ET distribution of each condition’s edges. An example for OGTT (0) is shown. (**K**) The comparison of ET distribution between each condition.

In this study, we attempted to use the edge types of a network to determine the difference between conditions: prior to and following OGTT, WT and *ob*/*ob*, and the metabolome and transcriptome. We used eight datasets: WT prior to OGTT, WT following OGTT, *ob*/*ob* prior to OGTT, and *ob*/*ob* following OGTT, for the metabolome and transcriptome, respectively. First, we inferred the network structure using mutual information prior to and following the OGTT in WT metabolome, referred to as the OGTT (0) and OGTT (4) networks, respectively (Fig. 1A,B). The nodes are connected by the edges, given that the mutual information^17^ between nodes is not significantly 0 in terms of statistical hypothesis test (*p* < 0.05, permutation test). Subsequently, the OGTT response network of the WT metabolome was defined as the difference between the OTTT (0) and the OGTT (4) network (Fig. 1B). Similarly, we defined the OGTT response network of the *ob*/*ob* metabolome as the difference between the OGTT (0) and the OGTT (4) networks. Then, we examined the abnormal OGTT response in *ob*/*ob* mice by comparing the OGTT response network between WT and *ob*/*ob*. The OGTT response networks of WT and *ob*/*ob* were compared in the transcriptome as well. The abnormal glucose metabolism in *ob*/*ob* mice was precisely characterized by the ETs. In the ET analysis, nodes were categorized according to the information from the KEGG database, and each ET was determined by both the node types at each end (Fig. 1C). The edges of the OGTT response networks were categorized into ETs, and the edges’ response to OGTT for each ET was compared between WT and *ob*/*ob*, as well as between metabolome and transcriptome data (Fig. 1D–G). Moreover, we obtained the distribution of ET by sorting the edges in a network of one condition into each ET, and summarized the difference network using the distribution of ET as an indicator (Fig. 1H–K). Consequently, it was found that the state of the network structure is reflected in the distribution of ETs, and that the characteristics of the state can be reduced to the distribution of ETs. The algorithmic procedure is described in “Materials and methods”.

### Distinct individual variations are present in WT and *ob*/*ob* mice

We had previously performed metabolomics and transcriptomics analyses of liver tissue from WT and *ob*/*ob* mice prior to (n = 11 for WT, n = 12 for *ob*/*ob*) and 4 h after (n = 12) an OGTT to determine the liver’s response to glucose perturbation^24^. We identified 262 metabolites and 22,463 genes from the metabolomics and transcriptomics analyses, respectively. OGTT (0) and OGTT (4) represent the conditions prior to and following the OGTT, respectively.

We performed principal component analysis to examine the outline of the data and found that WT formed a distinct group from *ob*/*ob* in the subspace of the first principal component, regardless of the OGTT, in the metabolome and transcriptome data (Fig. S1). This finding indicated that the metabolome and transcriptome data contained sufficient information to distinguish WT from *ob*/*ob*. In contrast, although the OGTT (0) and OGTT (4) in the WT and *ob*/*ob* groups could not be distinguished, minor variations in the metabolome were observed between individuals each of these groups. We observed minor variations in the OGTT (0) of WT and *ob*/*ob* groups and some large variations in the OGTT (4) of WT and *ob*/*ob* groups in the transcriptome. Thus, we found that the WT and *ob*/*ob* individuals were distinctly grouped, but individuals within these groups displayed variations in their metabolome and transcriptome.

### The OGTT response, based on the edges between WT and *ob*/*ob*, comprised considerable differences

**Figure 2:**
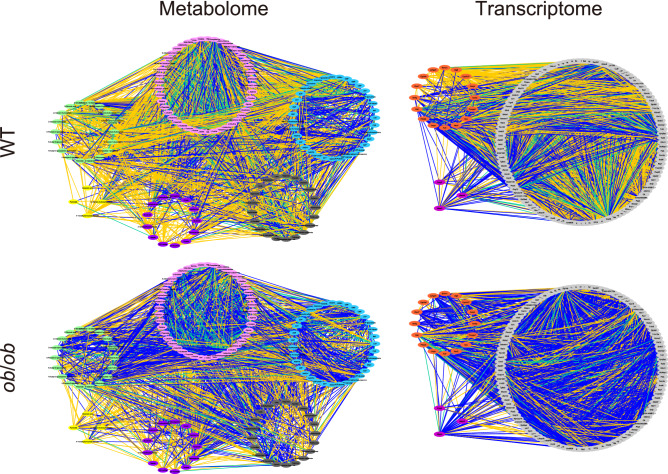
The OGTT response network. The OGTT response network of the metabolome (left column) and transcriptome (right column) in WT (upper row) and *ob*/*ob* (lower row). All of the 136 metabolites and the 136 genes randomly extracted from the 12,415 genes used for analysis were visualized. The blue, yellow, and green edge represents the edge that existed only in the OGTT (0) network, only in the OGTT (4) network, and that are common to both the networks, respectively.

In the metabolome, the mutual information of each pairwise metabolite was investigated under the following four conditions: WT OGTT (0), WT OGTT (4), *ob*/*ob* OGTT (0), and *ob*/*ob* OGTT (4). The network structure was inferred depending on whether the mutual information was positive or not. Mutual information between two nodes equaling zero indicates that they are statistically independent. Given that mutual information is statistically positive (*p* < 0.05, permutation test), the two nodes with estimated mutual information are connected by an edge (see “Materials and methods”, Fig. 2). Similarly, we examined the mutual information for the transcriptome in the four conditions. Our investigation revealed that the metabolomic and transcriptomic data used for network inference contained 136 metabolites and 12,415 genes that were quantified in the four previously mentioned conditions.

Subsequently, we examined the node degree distribution of the networks across the four distinct groups (Fig. S2). Our investigations revealed that the node degree distribution of OGTT (0) in the metabolome was not significantly different between WT and *ob*/*ob* (Table S1, Bonferroni corrected *p* < 0.05). In contrast, in OGTT (4), the networks for WT and *ob*/*ob* had significantly different distributions, with a wider tail and slightly narrower distribution, respectively. These findings suggested that the OGTT induced the network differences between WT and *ob*/*ob* and that the OGTT-induced changes in the structure of the metabolome network may be qualitatively distinct. In contrast, in the transcriptome, the node degree distribution in OGTT (0) was significantly different between WT and *ob*/*ob*, being bimodal in OGTT (0) in *ob*/*ob*, whereas that was unimodal in WT. In OGTT (4), the distribution of WT had a wider tail than the distribution of *ob*/*ob*, which was unimodal with a narrower tail; moreover, the difference between the two groups was significant. Thus, when compared with the metabolome, the transcriptomic analysis revealed a significant difference in the network structure between WT and *ob*/*ob* in OGTT (0). In contrast, the effect of the OGTT on the structural change in the transcriptome network was significantly different between WT and *ob*/*ob*, similar to the metabolome network. Furthermore, only one node of zero degree was found in the metabolome of WT OGTT (4) and *ob*/*ob* OGTT (4).

**Figure 3:**
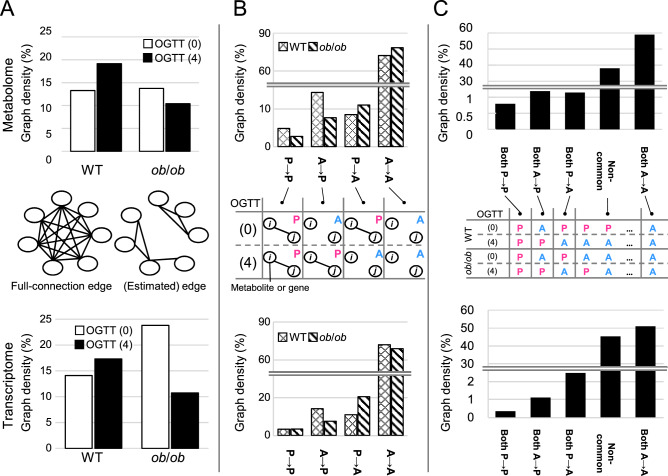
The edge amount quantification of each network. The graph density in each condition; (**A**) the graph density of the OGTT response network; (**B**) the comparison of the graph density of the OGTT response network between WT and *ob*/*ob*; (**C**) the graph density in the metabolome (upper panel) and transcriptome (lower panel). The middle panel in (**A**–**C**) are supplementary schematic diagrams that helps to interpret each graph. (**A**) The graph density of the OGTT (0) and OGTT (4) networks in WT and *ob*/*ob*. (**B**) The changes in the presence and absence of edges between the OGTT (0) and OGTT (4). “P → P,” “A → P,” “P → A,” and “A → A” stand for the edges, which are common to the OGTT (0) and OGTT (4) networks, the edges only existing in the OGTT (4) network, the edges only existing in the OGTT (0) network and the edges existing in neither OGTT (0) nor OGTT (4) networks, respectively. (**C**) The comparison between WT and *ob*/*ob* in the graph density of each edge explained in figure (**B**).

In this study, we defined full-connection edges as the edges of a network of fully connected nodes (Fig. 3A). The number of full-connection edges was equal to the maximum number of edges of the network, which is _*N*_C_2_, where *N* is the number of nodes in the network. The metabolome and transcriptome had a total of 9,180 and 77,059,905 full-connection edges, respectively. We compared the number of estimated edges to the number of full-connection edges to determine the graph density in networks with varying node sizes. In the metabolome, the graph density was 13.28% (1,219) for WT OGTT (0), 19.14% (1,757) for WT OGTT (4), 13.74% (1,261) for *ob*/*ob* OGTT (0), and 10.38% (953) for *ob*/*ob* OGTT (4) (Fig. 3A, upper panel). In the transcriptome, the graph density was 14.11% (10,874,216) for WT OGTT (0), 17.30% (13,334,125) for WT OGTT (4), 23.79% (18,330,266) for *ob*/*ob* OGTT (0), and 10.73% (8,271,366) for *ob*/*ob* OGTT (4) (Fig. 3A, lower panel). Additionally, the OGTT increased the number of edges in WT metabolome and transcriptome, whereas the opposite effect was found in *ob*/*ob*. This finding was consistent with the observation that the tails of the WT and *ob*/*ob* node degree distributions became wider and narrower, respectively, in both the metabolome and transcriptome (Fig. S2). Collectively, these observations indicate that the edge alterations induced by the OGTT resulted in a change in the tail of the node degree distribution.

Subsequently, we examined the differences between the WT and *ob*/*ob* in the presence and absence of edges between OGTT (0) and OGTT (4) (Figs. 1B, 3B). The OGTT response network was defined as the difference in network between OGTT (0) and OGTT (4). “P to P” denotes the edges that were shared by the OGTT (0) and OGTT (4) networks, “A to P” denotes the edges that were unique to the OGTT (4) network, “P to A” denotes the edges that were unique to the OGTT (0) network, and “A to A” denotes the edges existing in neither OGTT (0) nor OGTT (4) network. (in the figure, “to” is indicated by an arrow). Additionally, P to A, A to P, and P to P edges are considered as the OGTT response edges. We found that P to A and A to P edges existed in the metabolome and transcriptome of both WT and *ob*/*ob*. Thus, we calculated the net changes in the total number of edges for each individual. In the metabolome, the graph density of P to P edges was higher for WT than for *ob*/*ob*, suggesting that the former was possibly more resistant to glucose perturbation (Fig. 3B). Furthermore, we examined the number of the OGTT response edges that were identical between the WT and *ob*/*ob*, based on the graph density (Fig. 3C). More than half of all edges in both the metabolome and transcriptome were A to A in both groups. This implied that more than half of all edges were absent and were unchanged by the OGTT. Thus, we found that the same changes in the OGTT response between WT and *ob*/*ob* occurred in a limited part of the metabolome and transcriptome networks. Furthermore, with the exception of the A to A edges, the OGTT responses varied substantially between the WT and *ob*/*ob* groups.

### Difference in mutual information for most P to P edges is nonsignificant

As described previously, we determined the network structure using mutual information by examining statistical independence. The value of mutual information corresponding to the strength of edge connections can aid in understanding the network structure. However, the small sample size of the dataset made the value of the mutual information unreliable. Thus, we performed a statistical test to determine whether the mutual information values of two nodes were different between the networks (see “Materials and methods”).

For the P to P edges of the metabolome, our investigation revealed that there were 16 edges in WT (0.17% full-connection edges and 3.63% P to P edges) and 6 edges in *ob*/*ob* (0.07% full-connection edges and 2.41% P to P edges), with a significant difference for the OGTT (Table 1). In the transcriptome, there were 105,076 edges in WT (0.14% full-connection edges and 4.20% P to P edges) and 73,021 edges in *ob*/*ob* (0.09% full-connection edges and 2.85% P to P edges), with a significant difference for the OGTT. The ratio of P to P edges with different mutual information values was low in both WT and *ob*/*ob* in the metabolome and in the transcriptome. This result indicates that the major difference between the OGTT (0) and OGTT (4) networks was the structure rather than the strength of the connections. However, owing to the small sample size, it is possible that the number of edges with significant differences between OGTT (0) and OGTT (4) was underestimated.

**Table 1:** The number of the edges with the difference in the value of mutual information.

Metabolome		Transcriptome	
WT	16	WT	105,076
*ob*/*ob*	6	*ob*/*ob*	73,021

The number of the edges, which showed significantly different value of mutual information (*p* < 0.05, permutation test) in the P to P edges (described in Fig. 3B) in the metabolome (left) and transcriptome (right), respectively.

### ET characterizes the state of the network structure

Each node can be annotated with the biological property of the molecular species. Using node annotation, we defined the ET by categorizing the combination of annotations of pairwise connected nodes. Given that the properties of a molecular species are related to its function or role, the edges categorized as the same ET have similar properties and functions. We obtained the distribution of ETs for each condition based on the inferred network structure (see “Materials and methods”, Fig. 1H–K). Consequently, the statistical properties of the ET distribution will help to explain the relationship between network structures and biological phenomena.

**Figure 4:**
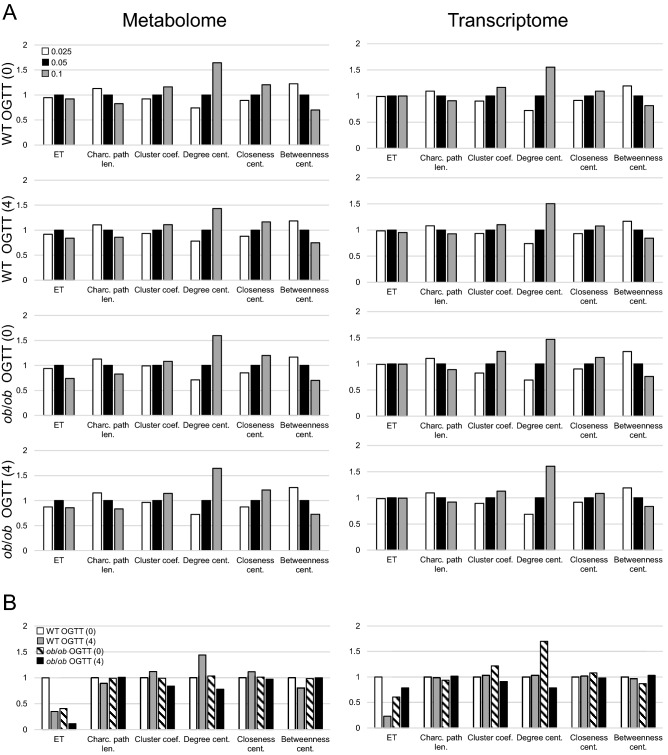
Comparison of the ET graph density and the conventional network statistics. The relative change of Spearman correlation coefficient of the ET graph density and the conventional network statistics against the *p* < 0.05 network in each condition (**A**) and of different conditions against the WT OGTT (0) network (**B**) in the metabolome (left column) and transcriptome (right column). Each value is normalized such that the value of *p* < 0.05 becomes one in (**A**) and that of WT OGTT (0) become one in (**B**). ET, Charc. path len., Cluster coef., Dgree cent., Closeness cent., and Betweenness cent. stand for absolute value of Spearman correlation coefficient of ET graph density, characteristic path length, cluster coefficient, the average of degree centrality, the average of closeness centrality, and the average of betweenness centrality, respectively.

One of the benefits of introducing ETs is the robust extraction of the network structure characteristics for comparison. To examine the robust extraction, we defined the graph density in which the counted edges are limited to a specific ET as the “ET graph density.” Although the threshold of the *p*-value affects the inferred network structure, when the threshold of the *p*-value is perturbed around 0.05, the Spearman correlation of ET graph density changes less than the conventional network statistics that are frequently used and estimated from network structure without using ETs, such as characteristic path length and cluster coefficient (Fig. 4A). This finding demonstrates that the rank of ET graph density is more robust than the conventional network statistics. Although the ET graph density is robust to perturbation of the *p*-value threshold, it highlights the difference between the network structure states. The ET graph density changes dynamically in response to the condition, whereas the conventional network statistics, other than the degree centrality, change very little compared with the ET graph density (Fig. 4B). However, degree centrality is not robust to perturbation of the *p*-value threshold. Thus, we concluded that the ET graph density enables robust network structure comparisons.

**Figure 5:**
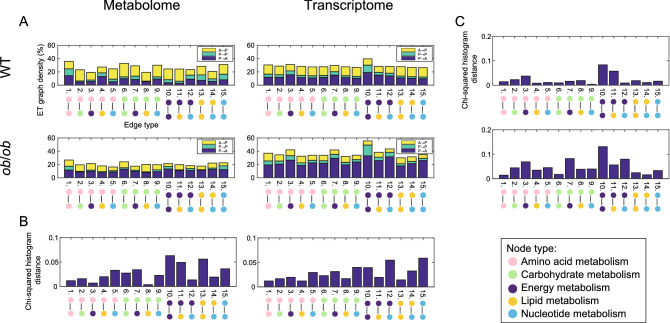
The ratio of OGTT response edge and comparison of the distribution of OGTT response edge in each edge type. (**A**) The ET graph density of each OGTT response edge (A to P, P to P, and P to A) in each ET in the metabolome (left column) and transcriptome (right column) of WT (upper row) and *ob*/*ob* (lower row), respectively. (**B**) The Chi-squared histogram distance of distributions of the ET graph density for the OGTT response edges between WT and *ob*/*ob*. (**C**) The Chi-squared histogram distance of distribution of the ET graph density for the OGTT response edges between the metabolome and transcriptome.

We examined the ET graph density of P to A, A to P, and P to P edges in the OGTT response network of WT and *ob*/*ob* (Fig. 5A, see also Fig. 1D–F). Based on the definitions in the KEGG database, metabolites and genes were annotated into 11 types, and we defined 66 ETs based on the combination of annotations for pairwise nodes (see “Materials and methods”). Additionally, the ET graph density of P to A, A to P, and P to P edges in each ET were examined in the metabolome and transcriptome (Fig. S3). Each ET is indicated by a number (No.), along with coupled annotations of the pairwise nodes that correspond to it. Coupled annotations for pairwise nodes are denoted by the initials of each node annotation separated by a hyphen (A: amino acid metabolism, C: carbohydrate metabolism, E: energy metabolism, L: lipid metabolism, and N: nucleotide metabolism). For example, the edge between amino acid metabolism and carbohydrate metabolism is represented as ET No. 2 (A–C).

Subsequently, we focused on 15 of the total of 66 ETs that were combinations of the major metabolite groups. Almost all ETs in the WT metabolome followed a similar pattern to the overall edges (Fig. 3B), with A to P edges having a higher ET graph density than P to A edges (Fig. 5A). ET No. 1 (A–A), 13 (L–L), and 15 (N–N) were notable in that their ET graph density of P to P edges was comparatively greater than that of other ETs. It would be of biological interest if associations between metabolites with similar biological characteristics were robust prior to and following the OGTT.

Almost all ETs in the *ob*/*ob* metabolome followed a similar pattern to the overall edges, with P to A edges having a higher ET graph density than A to P edges. ET No. 1 (A–A) and 15 (N–N) were distinctive in that the ET graph density of P to P edges was comparatively greater than that of other ETs. A high ET graph density for these ETs was also found for the WT metabolome. This suggested that the ETs unaffected by glucose perturbation were typically comparable between WT and *ob*/*ob*.

Subsequently, we examined the subtraction ranking of P to A from A to P of the ET. The difference in the subtraction corresponds to the increase in the net number of edges of OGTT (4) compared with OGTT (0). The smaller the difference, the smaller the increase in the number of edges in OGTT (4); if the difference was negative, the number of edges was lower in OGTT (4). The higher the difference was ranked in descending order, the more edges there were in OGTT (4). The lower the ranking, the more edges there were in OGTT (0). In the WT metabolome (Table S2), the top three ETs (in descending order) were ET No. 11 (E–L), 10 (E–E), and 2 (A–C) and the bottom three were ET No. 4 (A–L), 1 (A–A), and 12 (E–N). The difference in ET No. 4 (A–L), 1 (A–A), and 12 (E–N) was negative. Thus, the number of edges decreased following the OGTT. This result indicated an inverse relationship to the change in the overall edges following the OGTT. Given that edges reflect node interaction, the presence of ET No. 2 (A–C) in the top three indicates the possibility that the conversion between amino acid metabolism and carbohydrate metabolism was highly active in WT mice by the OGTT. Similar explanations existed for ET No. 11 (E–L) and 10 (E–E) in the top three ETs. If the conversions between energy metabolisms, as well as between energy metabolism and lipid metabolism were highly activated by the OGTT, this would be surprising given their associations with the OGTT. Similarly, glucose stimulation may have suppressed each conversion between amino acid metabolisms, between amino acid metabolism and lipid metabolism, and also between energy metabolism and nucleotide metabolism in OGTT (4) of WT mice.

In the ranking of the *ob*/*ob* metabolome (Table S3), the top three ETs (in descending order) were ET No. 8 (C–L), 2 (A–C), and 4 (A–L) and the bottom three were ET No. 10 (E–E), 15 (N–N), and 12 (E–N). The difference in ET No. 8 (C–L), 2 (A–C), and 4 (A–L) was positive. Thus, the number of edges increased following the OGTT. This result indicated an inverse relationship to the change in the overall edges by the OGTT. In both WT and *ob*/*ob*, ET No. 12 (E–N) appeared in the bottom three in and ET No. 2 (A–C) appeared in the top three. Given that an increase in the total number of edges in OGTT (4) indicates active metabolite conversion, the increase in the edge of ET No. 8 (C–L), 4 (A–L) in OGTT (4) suggested active conversion between carbohydrate metabolism and lipid metabolism, as well as between amino acid metabolism and lipid metabolism. However, it should be noted that the metabolites used in this study that are classified as lipid metabolism are short-chain fatty acids, rather than the long-chain fatty acids that accumulate as triglycerides. In contrast, the net decrease in the edges of ET No. 10 (E–E), 15 (N–N), and 12 (E–N) by the OGTT suggested the possibility that the conversion related to energy metabolism and nucleotide metabolism was inactive in OGTT (4) for the *ob*/*ob* metabolome.

In the WT transcriptome, some ETs followed a similar pattern to the overall edges, with A to P edges having a slightly higher ET graph density than P to A edges; others followed a different pattern. All ETs in the *ob*/*ob* transcriptome followed a similar pattern to the overall edges, with P to A edges having a higher ET graph density than A to P edges. ET No. 10 (E-E) was distinctive in that the ET graph density of P to P edges was comparably greater than that of other ETs in both the WT and *ob*/*ob* transcriptomes.

We examined the subtraction ranking of P to A from A to P of the ET in the transcriptome. In the WT transcriptome (Table S4), the top three ETs (in descending order) were ET No. 15 (N–N), 14 (L–N), and 9 (C–N) and the bottom 3 were ET No. 10 (E–E), 11 (E–L), and 3 (A–E). The top three, ETs No. 15 (N–N), 14 (L–N), and 9 (C–N), indicated the possibility that the transcriptome caused the changes in the amount of metabolic enzymes cooperating with each other between nucleotide metabolisms, and between lipid metabolism and nucleotide metabolism, and also between carbohydrate metabolism and nucleotide metabolism in conjunction between these metabolic pathways following the OGTT in WT mice. This result suggested that the gene expression related to nucleotide metabolism and glucose metabolism was possibly regulated by the OGTT as they are connected through the pentose phosphate pathway. In contrast, the bottom three, ETs No. 10 (E–E), 11 (E–L), and 3 (A–E), indicate a decrease in the cooperation of metabolic enzymes involved in energy metabolism. This result suggested that the coordination of gene expression regulation related to energy metabolism in OGTT (0) might have been disrupted by the transcriptional re-wiring mediated by the OGTT.

In the *ob*/*ob* transcriptome (Table S5), the top three ETs (in descending order) were ET No. 1 (A–A), 4 (A–L), and 13 (L–L) and the bottom three were ET No. 10 (E–E), 12 (E–N), and 7 (C–E). This result suggested that the cooperation of metabolic enzymes shifted away from energy metabolism to amino acid and lipid metabolism. The similarity between WT and *ob*/*ob* in the transcriptome was that ET No. 10 (E–E) appeared in the bottom three in descending order.

Certain ETs displayed a response to the OGTT that was consistent with the overall edges (Fig. 3A) in terms of increasing or decreasing the number of edges in the OGTT (4), whereas others displayed the opposite response (Table S6). By introducing ETs, each edge of the overall edges is grouped to a particular ET. Thus, we observed similar responses to the OGTT for WT and *ob*/*ob* mice in both the metabolome and transcriptome, but these could not be observed when overall edges were examined. ET No. 1 (A–A), 2 (A–C), 8 (C–L), and 12 (E–N) in the metabolome and ET No. 3 (A–E), 7 (C–E), 10 (E–E), 11 (E–L), and 12 (E–N) in the transcriptome showed similar responses to the OGTT between WT and *ob*/*ob*. This suggested that the OGTT response of *ob*/*ob* mice was not fully abnormal and the normal response, which was observed in WT mice, still occurred partially in *ob*/*ob* mice. Additionally, ET No. 1 (A–A), 3 (A–E), 4 (A–L), 7 (C–E), 10 (E–E), and 11 (E–L) in WT and ET No. 2 (A–C), 4 (A–L), and 8 (C–L) in *ob*/*ob* showed different response to the OGTT between the metabolome and transcriptome. Thus, one advantage of using ETs is that we can investigate similar properties shared by WT and *ob*/*ob* or different profile between the metabolome and transcriptome, which is difficult when individual edges are examined.

### Difference between WT and *ob*/*ob* reflects the distribution of OGTT response edges

We examined the divergence between distributions of P to A, A to P, P to P, and A to A edges for the OGTT response for each ET (Fig. 5B; see also Fig. 1D–G). The divergence between the distributions was measured by the Chi-squared histogram distance. This distance indicates the dissimilarity between two histograms, which is defined in the range between 0 and 1. When the Chi-squared histogram distance between two histograms is 0, the two histograms are completely equivalent. We compared the distribution of WT and *ob*/*ob* using the Chi-squared histogram distance.

In the metabolome, the top three ETs with the largest Chi-squared histogram distance between WT and *ob*/*ob* were ET No. 10 (E–E), 13 (L–L), and 11 (E–L), all of which were involved in energy and lipid metabolism (Table S7). This finding indicated that the distributions of edges’ responses to the OGTT in these ETs were considerably different between WT and *ob*/*ob*. Indeed, it has already been reported that lipid synthesis is abnormally enhanced in *ob*/*ob* mice^27,28^. Additionally, it has been reported that *ob*/*ob* mice have abnormal energy metabolism, including decreased energy expenditure^25,29^, decreased mitochondrial respiration^30^, and switching of the energy substrate from glucose to fatty acid^31^.

The top three ETs with the smallest Chi-squared histogram distance between WT and *ob*/*ob* were ET No. 8 (C–L), 3 (A–E), and 1 (A–A). Two of these were involved in amino acid metabolism, suggesting that the response of edges involved in amino acid metabolism to the OGTT was similar in WT and *ob*/*ob*. The small Chi-squared histogram distance found for ET No. 8 (C–L) is paradoxical because de novo lipid synthesis is increased and carbohydrate to lipid conversion is also active in *ob*/*ob* mice^27,28,32^, even though the lipid metabolites in this study were not long-chain fatty acids. This counterintuitive result obtained by our methodology is exciting, as it may have yielded new insight.

In the transcriptome, the top three ETs with the largest Chi-squared histogram distance between WT and *ob*/*ob* were ET No. 15 (N–N), 12 (E–N), and 9 (C–N) (Table S7). The Chi-squared histogram distance found for ET No. 10 (E–E) was slightly smaller than ET No. 9 (C–N). The top three ETs with the smallest Chi-squared histogram distance between WT and *ob*/*ob* were ET No. 1 (A–A), 4 (A–L), and 13 (L–L). ETs involved in energy metabolism were found in the top three largest Chi-squared histogram distances, whereas ETs involved in amino acid metabolism were found in the top three smallest Chi-squared histogram distances in both the metabolome and transcriptome. The pattern of such relationships to annotation would be instructive in revealing the points of convergence and divergence between WT and *ob*/*ob*. Indeed, abnormal energy metabolism has been reported in *ob*/*ob* mice, including altered abundance of oxidative phosphorylation proteins^30^.

The Chi-squared histogram distance found for ET No. 10 (E-E) was large in both the metabolome and transcriptome, indicating that the response of edges to the OGTT related to energy metabolism was different in the metabolome and transcriptome between WT and *ob*/*ob*. Thus, there is a possibility that the substantial difference in transcriptional regulation in response to the OGTT between WT and *ob*/*ob* mice in energy metabolism may be reflected in the metabolome.

ET No. 1 (A–A) appeared in the top three ETs with the smallest Chi-squared histogram distance in both the metabolome and transcriptome, suggesting that the response of edges related to amino acid metabolism to the OGTT was slightly different between WT and *ob*/*ob* in both the metabolome and transcriptome. The transcriptional regulation and effect of the OGTT on the metabolome related to amino acid metabolism should differ slightly between WT and *ob*/*ob* mice owing to an indirect impact of the OGTT on amino acid metabolism.

The scatter plot of the Chi-squared histogram distance between WT and *ob*/*ob* for the metabolome and transcriptome is shown in Fig. S4. We found that the Chi-squared histogram distance of ET No. 10 (E–E) was large, and that of ET No. 1 (A–A) was small in both the metabolome and transcriptome. Additionally, we found that the Chi-squared histogram distances associated with amino acid metabolism were small and so were located near the origin of the scatter plot. In contrast, the Chi-squared histogram distances associated with energy metabolism were large and far from the origin. Finally, we found that the Chi-squared histogram distance associated with nucleotide metabolism in the transcriptome was typically greater than that of the metabolome and that the distance associated with the lipid metabolism in the metabolome was typically greater than that of the transcriptome.

### Regulation of the relationship for OGTT response between the metabolome and the transcriptome described by the distribution of ET

We examined the Chi-squared distance between the distributions of the OGTT response edges of the metabolome and the transcriptome for each ET. The changes in the graph density were similar between the metabolomes and transcriptomes in WT and *ob*/*ob* (Fig. 3A), whereas the Chi-squared distances varied among ETs in WT and *ob*/*ob* (Fig. 5C). The top three ETs with the smallest distances in WT and *ob*/*ob* were ET No. 9 (C–N), 4 (A–L), and 12 (E–N), and ET No. 1 (A–A), 14 (L–N), and 6 (C–C), respectively (Table S8). The small distances suggest the existence of regulatory relationships between the metabolome and the transcriptome for the ETs in WT and *ob*/*ob* mice. Additionally, we found that the Chi-squared distance between *ob*/*ob* was greater than that between WT for each ET (Fig. S5). This observation implied that the regulatory relationship between the metabolome and transcriptome was disrupted in *ob*/*ob* mice in comparison to WT mice.

### Reduction of characteristics of OGTT response edges by multidimensional scaling

**Figure 6:**
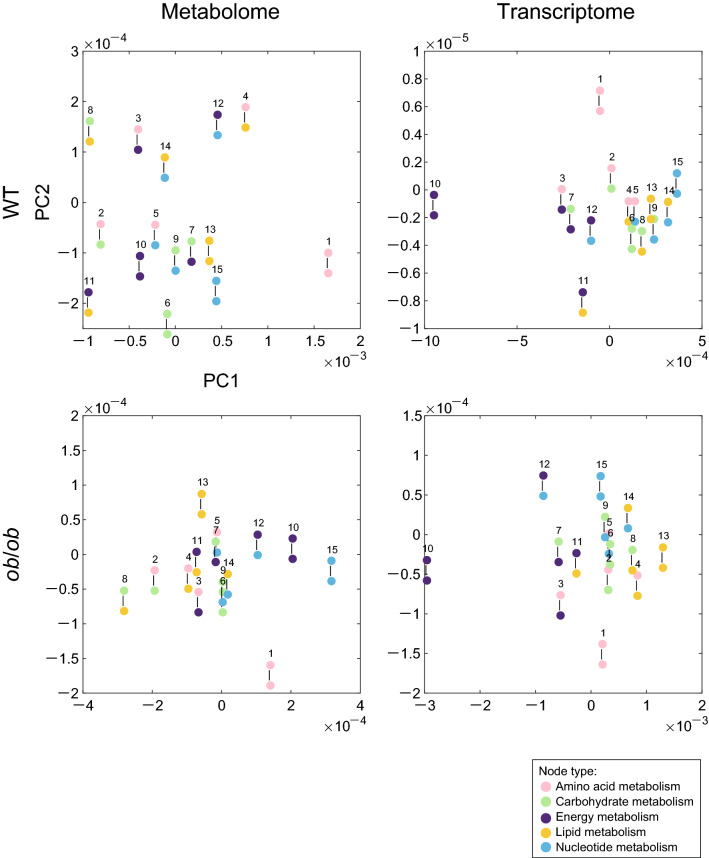
The MDS plot for the ETs. ETs are arranged according to Chi-squared histogram distance of the distributions of the OGTT response edges. The metabolome of WT (left upper panel) and *ob*/*ob* (left lower panel), the transcriptome of WT (right upper panel) and *ob*/*ob* (right lower panel).

Multidimensional scaling (MDS) is a technique for visualizing the level of similarities of pairwise samples in a dataset that creates a map displaying the relative positions. We visualized the similarity between ETs, which are represented by the Chi-squared distance in the distributions of the OGTT response edges, such as P to A, A to P, and P to P, in a two-dimensional plot using MDS (Fig. 6). ET No. 1 (A–A) was located further from the other ETs in all four MDS plots, suggesting that the edges of ET No. 1 (A–A) indicate a distinct role in the OGTT response. Additionally, ET No. 10 (E–E) is located further from the other ETs, except for the WT metabolome, suggesting that the edges of ET No. 10 (E–E) indicate a distinct role in the OGTT response apart from in the WT metabolome. Additionally, we found that the MDS map of WT and *ob*/*ob* was more similar for the transcriptome than for the metabolome, which is consistent with the fact that the variance of the Chi-squared distance for the metabolome is greater than that for the transcriptome (Fig. 5B). Thus, an MDS map can be helpful to extract the characteristics of ET and reduce the information.

### The distribution of ETs contains reduced information on the state of the network structure

**Figure 7:**
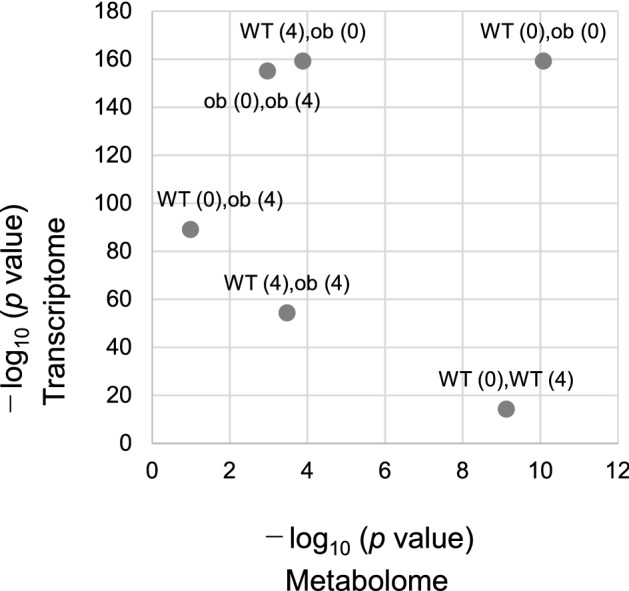
The scatter plot of − log_10_ (*p*-value) between the metabolome and transcriptome. The scatter plot of the − log_10_ (Bonferroni corrected *p*-value) between the metabolome and transcriptome. WT (0), WT (4), ob (0), and ob (4) represent WT OGTT (0), WT OGTT (4), *ob*/*ob* OGTT (0), and *ob*/*ob* OGTT (4), respectively.

The two-sample Cramér–von Mises test^33^ is a statistical hypothesis test in which the null hypothesis is that the two samples are drawn from the same distribution. It was used to determine whether the two ET distributions were distinct. We assumed that the ETs followed an identical distribution independently and used the two-sample Cramér–von Mises test to determine the difference between the distributions of ETs for WT OGTT (0), WT OGTT (4), *ob*/*ob* OGTT (0), and *ob*/*ob* OGTT (4) in the metabolome and transcriptome (Table 2, Bonferroni corrected *p* < 0.05; see also Fig. 1H–K). All pairwise distributions were significantly different from each other, except for the pairing of WT OGTT (0) and *ob*/*ob* OGTT (4) in the metabolome data. These findings suggest that the state of the network structure is reflected in the distribution of ETs, and that the characteristics of the state can be reduced to the distribution of ET. Additionally, the possibility of a difference between WT OGTT (0) and *ob*/*ob* OGTT (0) was greater in the metabolome and transcriptome than that for the other pairs (Fig. 7). Likewise, the possibility of a difference between WT OGTT (4) and *ob*/*ob* OGTT (4) was lower than that for the majority of other metabolomics and transcriptomic variables. This finding suggested that the glucose perturbation decreased the possibility of a difference between WT and *ob*/*ob*. In other words, this result might suggest that glucose perturbation (by the OGTT) and the mechanism by which the liver maintains glucose homeostasis reduced the difference in the hepatic metabolome and transcriptome between WT and *ob*/*ob* mice compared with steady-state conditions, which were WT OGTT (0) and *ob*/*ob* OGTT (0), in terms of the biological categorization of inferred molecular relationship based on mutual information. Although the possibility of a difference between WT OGTT (0) and WT OGTT (4) was similar to that between WT OGTT (0) and *ob*/*ob* OGTT (0) in the metabolome, the possibility of difference between WT OGTT (0) and WT OGTT (4) was markedly reduced compared with the possibility of a difference between WT OGTT (0) and *ob*/*ob* OGTT (0) in the transcriptome. In contrast, although the possibility of difference between *ob*/*ob* OGTT (0) and *ob*/*ob* OGTT (4) was similar to that between WT OGTT (0) and *ob*/*ob* OGTT (0) in the transcriptome, the possibility of difference between *ob*/*ob* OGTT (0) and *ob*/*ob* OGTT (4) in the metabolome was markedly reduced when compared to the possibility of difference between WT OGTT (0) and *ob*/*ob* OGTT (0) in the metabolome. In other words, following glucose perturbation (by the OGTT), the metabolome of WT and the transcriptome of *ob*/*ob* could be as dissimilar as WT and *ob*/*ob* under steady-state conditions (WT OGTT (0) and *ob*/*ob* OGTT (0)), respectively. Thus, the metabolome of *ob*/*ob* and the transcriptome of WT were relatively similar compared with the steady-state, following glucose perturbation. These observations collectively suggested that WT and *ob*/*ob* mice were affected by glucose perturbation with changes in the metabolome and transcriptome, respectively. Conversely, the transcriptome of WT mice and the metabolome of *ob*/*ob* mice are suggested to be less affected by the OGTT. Consequently, the positional relationship between “WT OGTT (0) and WT OGTT (4)” and “*ob*/*ob* OGTT (0) and *ob*/*ob* OGTT (4)” was symmetrical with respect to a line with a positive slope. The positional relationship between “WT OGTT (0) and *ob*/*ob* OGTT (4)” and “WT OGTT (4) and *ob*/*ob* OGTT (0)” was symmetric with respect to a line with a negative slope. Thus, the distribution of ETs contains reduced information on the state of the network structure.

**Table 2:** The distance of A^2^ statistics and *p*-values.

Metabolome					Transcriptome				
A^2^					A^2^				
	WT (0)	WT (4)	*ob*/*ob* (0)	*ob*/*ob* (4)		WT (0)	WT (4)	*ob*/*ob* (0)	*ob*/*ob* (4)
WT (0)		25.745654	27.9798667	3.33705359	WT (0)		37.932303	1134.66383	211.002001
WT (4)			7.21091626	7.77674533	WT (4)			1181.80553	130.839894
*ob*/*ob* (0)				11.1609826	*ob*/*ob* (0)				363.202317
*ob*/*ob* (4)					*ob*/*ob* (4)				
*p*-value					*p*-value				
	WT (0)	WT (4)	*ob*/*ob* (0)	*ob*/*ob* (4)		WT (0)	WT (4)	*ob*/*ob* (0)	*ob*/*ob* (4)
WT (0)		7.5057E-10	8.3855E-11	0.10291111	WT (0)		4.8327E-15	< 1.0E-16	< 1.0E-15
WT (4)			0.00013157	0.0003385	WT (4)			< 1.0E-16	< 1.0E-15
*ob*/*ob* (0)				0.00106993	*ob*/*ob* (0)				< 1.0E-15
*ob*/*ob* (4)					*ob*/*ob* (4)				

The left and right column represent the metabolome and transcriptome, respectively. The upper and lower table are the A^2^ statistics and Bonferroni corrected *p*-values. In both the tables, WT (0), WT (4), *ob*/*ob* (0), and *ob*/*ob* (4) stands for WT OGTT (0), WT OGTT (4), *ob*/*ob* OGTT (0), and *ob*/*ob* OGTT (4), respectively.

## Discussion

In this study, we inferred the OGTT response networks using statistical independence for WT and *ob*/*ob* mice in the metabolome and transcriptome to determine the difference in response to the OGTT between WT and *ob*/*ob* mice. Our results showed that glucose perturbation by the OGTT increased the number of metabolome and transcriptome network edges in WT, but the number was decreased in *ob*/*ob* (Fig. 3A). Furthermore, we observed that the majority of the edge responses to the OGTT for the metabolome and transcriptome were different between WT and *ob*/*ob*, except for the A to A edges (Fig. 3C). Subsequently, we analyzed the ETs and found the following two points: (1) There was a similar OGTT response in terms of ET between WT and *ob*/*ob* in the metabolome and transcriptome data (Table S6); and (2) the OGTT responses were different in terms of ET between the metabolome and transcriptome, even though the responses of the overall edges were similar. These findings could not be obtained by analyses without introducing ETs such as the analysis of node degree distribution and overall edges.

Owing to the complexity and unreliability of the results obtained from inferring the network structure from a dataset with a small sample, we chose to use the results given using ET. Additionally, comparing the different network structures is quite challenging. However, our study is novel in that it uses information on ET to reduce the amount of information about the state of the network structure and to compare between states of network structure. We used a simple “top three” selection to determine the remarkable ETs in the evaluation of the edges’ response to OGTT (Fig. 5A, Tables S2–S5) or the comparison of the edges’ response between WT and *ob*/*ob* or between the metabolome and the transcriptome (Fig. 5B–C, Tables S7, S8). However, there is a possibility that we can more reasonably select remarkable ETs. In future work, we will aim to develop a procedure to select remarkable ETs.

Kokaji et al.^22^ previously examined the OGTT response in WT and *ob*/*ob* mice, but their analysis was based on the average of each molecule, which differs from our definition of “OGTT response network.” However, our results corroborate their findings, as we found that the OGTT response network were considerably different between WT and *ob*/*ob*. Additionally, both studies found that a small number of edges appeared in both WT and *ob*/*ob*, whereas a large number of edges were unique to either WT or *ob*/*ob*.

The nodes were annotated with information limited to the metabolic pathways in the KEGG database, which enabled us to compare the metabolome and transcriptome using the ET categorization. Annotation of the metabolome is also possible using the Human Metabolome Database. A single metabolite is annotated into multiple metabolic pathways in the KEGG database, whereas the Human Metabolome Database annotates metabolites without redundancy. Therefore, the Human Metabolite Database would have been a better choice for this study if we were not making comparisons between the metabolome and transcriptome. Except for metabolic pathways, the genes in the KEGG database are lacking for the transcriptome and do not cover all datasets. TRANSFAC and RegNetwork provide information about transcription factors, but their annotation coverage is insufficient for genes. Gene ontology (GO) is the most complete source of gene annotation. However, the use of GO requires reformulation of the hierarchical ontology for the ET. Consequently, we chose to use the KEGG database annotation, allowing comparison between the transcriptome and metabolome. Cellular systems consist of several layers, including the metabolome and transcriptome, as well as the epigenome and proteome. We emphasize that our method of analyzing networks using common ETs has great potential for network analysis across multiple omics layers.

In biological research, a dataset is usually preprocessed by averaging across individuals for each molecular species. The averaging preprocess is reasonable and simplifies analysis, given our interest in the average behavior of phenomena. However, the averaging preprocess carries the disadvantage of losing information about variations between individuals, which is necessary to establish relationships between molecules. Therefore, we inferred the network structure using individual information based on statistical independence between molecular species. Linear correlations, such as the Pearson correlation, are frequently used to examine relationships between molecular species; however, they are incapable of detecting nonlinear relationships. In contrast, mutual information^17^ enables the detection of statistical independence by evaluating nonlinear relationships, including linear relationships. Conditional independence can yield a more precise network structure because of the exclusion of the effects of other molecular species, which indirectly affects the relationships between targeted pairwise molecular species. However, detecting conditional independence is difficult in datasets with a high number of dimensions and a small sample size. Additionally, the computational burden scales exponentially with the number of dimensions in the datasets. Therefore, we examined statistical independence to infer network structure in this study and, in the future, plan to continue to adopt conditional independence to infer network structure.

Owing to the high cost of biological experiments, the dataset had a small sample size, necessitating the use of the permutation test to determine statistical independence. Thus, we inferred the network structure and omitted the intensity of connections because of the low reliability of the mutual information value.

The small sample size of the dataset reduces the reliability of the inferred whole network structure, indicating that an edge-by-edge comparison of whole network structures is not robust. Additionally, it is critical to define a quantitative comparison metric for network structures, which is not easy. Information about the entire network structure, however, can be reduced to the distribution of ETs, which is insensitive to changes in edges. Additionally, we demonstrated that this reduced information enables the comparison of two states of network structure, corresponding to the WT and *ob*/*ob* OGTT responses, or the metabolome and transcriptome, respectively. Collectively, these findings suggest that the reduced information obtained from the distribution of ET can be used as a “fingerprint,” indicating characteristics of the state of network structure. The fingerprint is analogous to the bag-of-words model used in the field of text analysis. To summarize, the bag-of-words model reduces the complexity of a text by counting the occurrences of words in documents. In this method, even though documents are characterized using word counts and the information about the text structure is reduced, the model is useful for document classification and other purposes. However, additional research is required to show the advantage of the fingerprint by applying it to a variety of biological phenomena.

## Materials and methods

### Mouse studies

Previously published mouse liver samples were used for this study^24^. Briefly, ten-week-old male C57BL/6 wild-type and *ob*/*ob* mice were purchased from Japan SLC Inc. After overnight fasting (16 h), mice were administered 2 g/kg body weight of glucose orally. After 4 h from the experiments, mice were sacrificed by cervical dislocation and the liver (whole or left lateral lobe) was dissected and immediately frozen in liquid nitrogen. Similarly, the livers from overnight fasted (16 h) mice were dissected and frozen as a control. The frozen liver was pulverized with dry ice to a fine powder with a blender and separated into tubes for omic analysis (metabolomics, and transcriptomics). We used twelve WT and twelve *ob*/*ob* mice following oral glucose administration, and eleven WT and twelve *ob*/*ob* mice in a control group. The metabolomics and transcriptomic data of all the samples were reported in previous study^24^ and the metabolites and genes used for our analysis were expanded to include other metabolic pathways in addition to central carbon metabolism. All methods were carried out in accordance with relevant guidelines and all the mouse experiments were approved by the animal ethics committee of The University of Tokyo. This study is reported in accordance with ARRIVE guidelines.

### Principal component analysis

We performed the principal component analysis for centralized datasets of the metabolome and transcriptome, which were reformulated as the fold changes for the average of each molecular species.

### Procedure of network inference and ET identification

Suppose that ${X}_{i}$ are a random variable, which correspond to the amount of molecular species *i*, such as gene expression and metabolite concentration, where $i=1,\cdots ,m$ and $m$ is the number of molecular species. ${V}_{i}$ represents a vertex of network, corresponding to molecular species *i*. ${E}_{ij}=\{\mathrm{0,1}\}$ represents the edge between ${V}_{i}$ and ${V}_{j}$. If ${E}_{ij}=1$, the ${V}_{i}$ and ${V}_{j}$ are connected each other, otherwise they are not connected. ${p}_{0}$ represents the threshold on *p*-value to determine edge existence. The procedure of part of network inference is as follows.

**Step 1**: evaluate the *p*-value by statistical hypothesis test, whose null hypothesis is $I\left({X}_{i};{X}_{j}\right)=0$, for $i>j, i,j=1,\cdots m$. The *p*-value of ${E}_{ij}$ is denoted by ${p}_{ij}$.

**Step 2**: if ${p}_{ij}<{p}_{0}$, ${E}_{ij}=1$. Otherwise ${E}_{ij}=0$.

After the network structure is determined by network inference, the ET is identified. Suppose that the set of node type is denoted by ${N}_{type}=\{{n}_{1},\cdots ,{n}_{s}\}$, where ${n}_{k},k=1,\cdots ,s$ and *s* represent a node type and the number of node types, respectively. ${N}_{type}$ is determined based on database and one can set it arbitrarily in accordance with the aim. ${V}_{type,i}$ represents the node type of ${V}_{i}$. If the node type of molecular spices *i* is ${n}_{k}$, ${V}_{type,i}={n}_{k}$. Next, suppose that the set of ET is denoted by ${E}_{type}=\{{e}_{1},\cdots ,{e}_{t}\}$, where ${e}_{k},k=1,\cdots ,t$ and *t* represent an ET and the number of ETs, respectively. The ET ${e}_{k}$ *t* does not exceed the $\frac{s\left(s-1\right)}{2}+s$. ${E}_{type,ij}$ represents the edge type of ${E}_{ij}$. If the edge type of ${E}_{ij}$ is ${e}_{k}$, ${E}_{type,ij}={e}_{k}$. The procedure of part of ET identification is as follows.

**Step 3**: identify the node type ${V}_{type,i}$ from ${N}_{type}$ for $i=1,\cdots ,m$, which is determined by annotation of molecular species *i* based on database.

**Step 4**: identify the ET ${E}_{type,ij}$ from ${E}_{type}$ for all pairs of *i* and *j* satisfying ${E}_{ij}=1$, according to the pair of node type ${V}_{type,i}$ and ${V}_{type,j}$.

Finally, we obtain the distribution of ET by counting the appearance of each ET ${e}_{k}$, for ${E}_{type,ij}$ of all pairs of *i* and *j* satisfying ${E}_{ij}=1$. Note that there is a possibility that multiple node types assign to one node, for instance, ${V}_{type,i}=\{{e}_{k},{e}_{l}\}$. In the case of multiple node types, the ETs corresponding to all pairwise combination of edge node between ${V}_{type,i}$ and ${V}_{type,j}$ assign to ${E}_{type,ij}$. And one counts the appearance of each ET in ${E}_{type,ij}$, which is equally weighted by $1/{\#E}_{type,ij}$, where ${\#E}_{type,ij}$ represents the number of ETs contained in ${E}_{type,ij}$.

### OGTT response network definition

The OGTT response network is defined as the difference of network between OGTT (0) and OGTT (4). The edges in the OGTT response network are denoted by four types, according to their presence or absence in the network of OGTT (0) and OGTT (4): “P to P” denotes the edges that are shared by the OGTT (0) and OGTT (4) networks, “A to P” denotes the edges that are unique to the OGTT (4) network, “P to A” denotes the edges that are unique to the OGTT (0) network, and “A to A” denotes the edges existing in neither OGTT (0) nor OGTT (4) network. Additionally, P to A, A to P, and P to P edges are referred to as the OGTT response edges. The ET in the OGTT response network is identified in the same way of step3 and step4 in the procedure above.

### Calculation of the mutual information

The mutual information of two random variables *X* and *Y* is defined as

$$I\left(X,Y\right)=\iint p(x,y)\mathrm{log}\frac{p(x,y)}{p(x)p(y)}dxdy$$

where *p*(*x*,*y*) is the joint probability distribution of *X* and *Y*, and *p*(*x*) and *p*(*y*) are marginal probability distribution of *X* and *Y*, respectively.

The estimation of joint probability distribution of *X* and *Y* from the dataset is required in order to calculate the mutual information. We estimated the joint probability distribution using B-spline function^17^. The spline order *k* was set to *k* = 3 and the number of bin *M* was determined in each combination of *X* and *Y*^34^. The metabolome and transcriptome data were standardized and subjected to calculation of the mutual information.

### Statistical hypothesis test on statistical independence

The permutation test is performed under the null hypothesis that the mutual information $I\left(X;Y\right)$ is 0^35^. If and only if $I\left(X;Y\right)=0$, *X* and *Y* are statistically independent. The sample of null distribution is generated by permutation of original data *X*. We obtained the empirical null distribution by the random sampling, where the sample size was 500 in each permutation test. The *p*-value is obtained by the ratio of the number of samples, which are larger than the value of mutual information $I\left(X;Y\right)$, to the sample size of empirical null distribution.

### Statistical hypothesis test on the difference of values of mutual information

To examine whether the values of the mutual information are different between prior to and following the OGTT, we performed the permutation test under the null hypothesis and alternative hypothesis described in following formula

$$\left\{\begin{array}{c}{H}_{0}:{I}_{OGTT (0)}\left(X;Y\right)={I}_{OGTT (4)}(X;Y)\\ {H}_{1}:{I}_{OGTT (0)}\left(X;Y\right)\ne {I}_{OGTT (4)}(X;Y)\end{array}\right.$$

The sample size was 500 to generate null distribution empirically in each permutation test.

### Network visualization

Cytoscape^36^ was used for network visualization. We used the method that is as non-redundant as possible for node categorization in visualization. The metabolites were categorized using the information of the Human Metabolome Database^37–40^ in the metabolome. In the transcriptome, the genes whose OS and BS fields include both Mus musculus in the factor.dat file in TRANSFAC^41^ were categorized as transcription factor. The genes which were registered in RegNetwork^42^ as Type: regulator, Organism: mouse, Evidence: Experimental, Confidence: High were also categorized as transcription factor. The genes which were registered to the pathway included in “1. Metabolism” in KEGG PATHWAY database^43–45^ were categorized as metabolic enzyme. ENSMUSG00000017715 (Pgs1) and ENSMUSG00000020593 (Lpin1) categorized to both transcription factors and metabolic enzymes, but were classified as transcription factors. The genes not categorized to neither transcription factor and metabolic enzyme were categorized as Others.

### Edge type categorization

For node categorization, we used following 11 calcifications in “1. Metabolism” of KEGG PATHWAY database: 1.1 Carbohydrate metabolism, 1.2 Energy metabolism, 1.3 Lipid metabolism, 1.4 Nucleotide metabolism, 1.5 Amino acid metabolism, 1.6 Metabolism of other amino acids, 1.7 Glycan biosynthesis and metabolism, 1.8 Metabolism of cofactors and vitamins, 1.9 Metabolism of terpenoids and polyketides, 1.10 Biosynthesis of other secondary metabolites, 1.11 Xenobiotics biodegradation and metabolism. The metabolites or genes which belong to the pathway included in each classification were categorized into these 11 types. Next, 66 edge types connecting between 11 node types were defined.

### Calculation of graph density

The graph density *D* and the ET graph density ${D}_{ET}$ were calculated by flowing formulas.

$$D=\frac{2E }{N\left(N-1\right)}$$

$${D}_{ET}=\frac{2{E}_{ET} }{{N}_{ET}\left({N}_{ET}-1\right)}$$

where *E* is the number of estimated edges, ${E}_{ET}$ is the number of edges that estimated and categorized into each ET, *N* is the number of the node in the whole graph and ${N}_{ET}$ is the number of the node in the subgraph categorized into each ET.

### Chi-squared histogram distance

The Chi-squared histogram distance between the histograms of *P* and *Q* is defined by

$${\chi }^{2}\left(P,Q\right)=\frac{1}{2}\sum_{i}\frac{{\left({P}_{i}-{Q}_{i}\right)}^{2}}{\left({P}_{i}+{Q}_{i}\right)}$$

where the subscript *i* indicates the *i*-th bin of the histogram of *P* or *Q*, *P*_*i*_ and *Q*_*i*_ indicate the frequency of each *i*-th bin.

### MDS plot

Torgerson scaling method^46^ determines the coordinate of ${x}_{i}$ so as to minimize

$$\sum_{i,j}{\left({z}_{ij}-\sum_{m}{x}_{im}{x}_{jm}\right)}^{2}$$

where ${z}_{ij}=\frac{1}{2}\left({d}_{io}^{2}+{d}_{jo}^{2}-{d}_{ij}^{2}\right)$, ${d}_{ij}$ is the distance between the coordinates of ${x}_{i}$ and ${x}_{j}$, and o indicates the coordinate of the origin. In this study, ${d}_{ij}^{2}$ corresponds to Chi squared histogram distance between distributions of ET.

### Implementation

All analysis was implemented by MATLAB 2016b (The Mathworks Inc.).

## Supplementary Information

Supplementary Information.

## Data Availability

Sequencing data measured in this study have been deposited in the DNA Data Bank of Japan Sequence Read Archive (DRA) (www.ddbj.nig.ac.jp/) under the accession no. DRA008416 and DRA012292.
