# Supplementary material for: Comparison of hepatic responses to glucose perturbation between healthy and obese mice based on the edge type of network structures
Source: Sci Rep. 2023 Mar 23;13:4758. doi: 10.1038/s41598-023-31547-2 (PMC10036622; doi:10.1038/s41598-023-31547-2)
Supplement: Supplementary file 1 — Supplementary Information. [file 41598_2023_31547_MOESM1_ESM.docx]

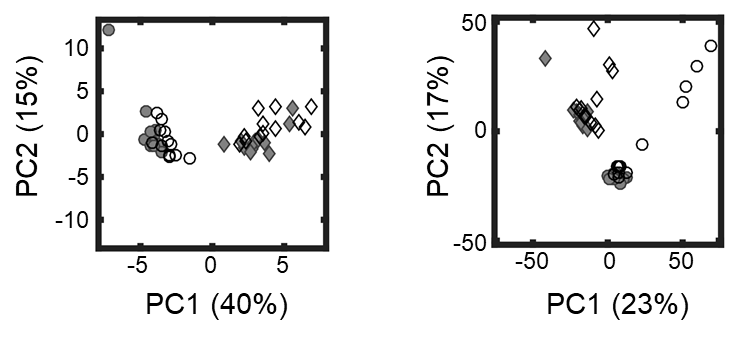


## **Fig. S1 PCA score plot of metabolome and transcriptome data.**

The PCA score plot of the metabolome (left) and transcriptome (right). Circle and diamond marker represents WT and *ob*/*ob*, respectively. Closed and opened marker represents OGTT (0) and OGTT (4), respectively. The contribution of each principal component was indicated along the axis.


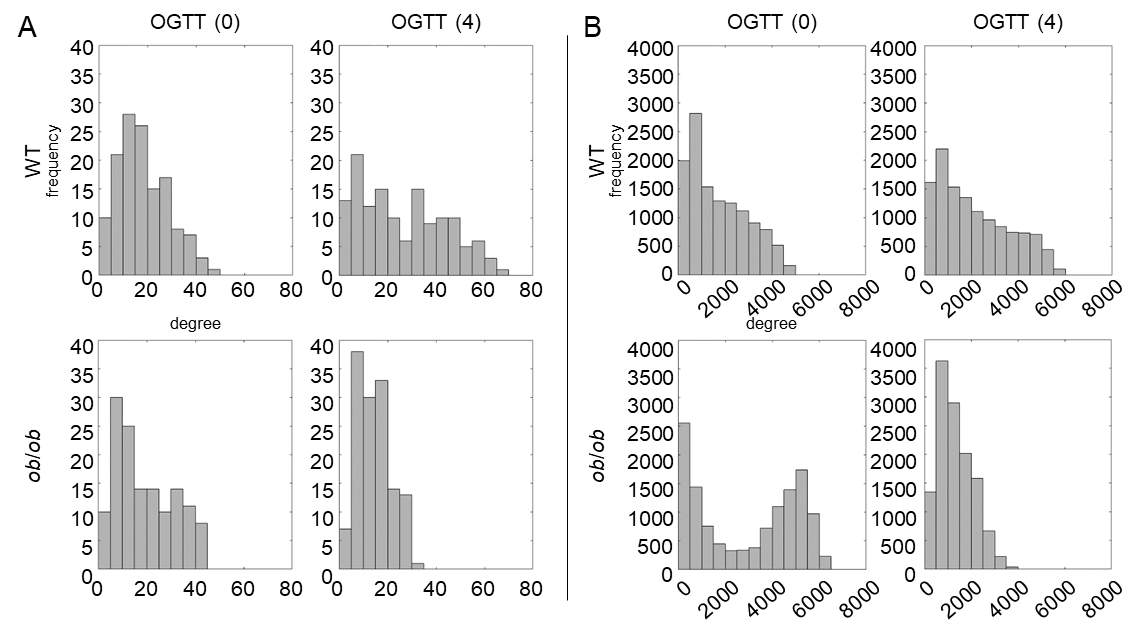


## **Fig. S2 The degree distribution of each inferred network.**

(A) The node degree distribution in the metabolome. The mean and standard deviation of the degree distribution in each network was indicated in the following: WT OGTT (0): 17.93±10.30, WT OGTT (4): 25.84±17.32, *ob*/*ob* OGTT (0): 18.54±12.03, *ob*/*ob* OGTT (4): 14.01±6.92. (B) The node degree distribution of the networks in the transcriptome. The mean and standard deviation of the degree distribution in each network was indicated in the following: WT OGTT (0): 1751.79±1226.95, WT OGTT (4): 2148.07±1509.63, *ob*/*ob* OGTT (0): 2952.92±2091.78, *ob*/*ob* OGTT (4): 1332.48±734.04.


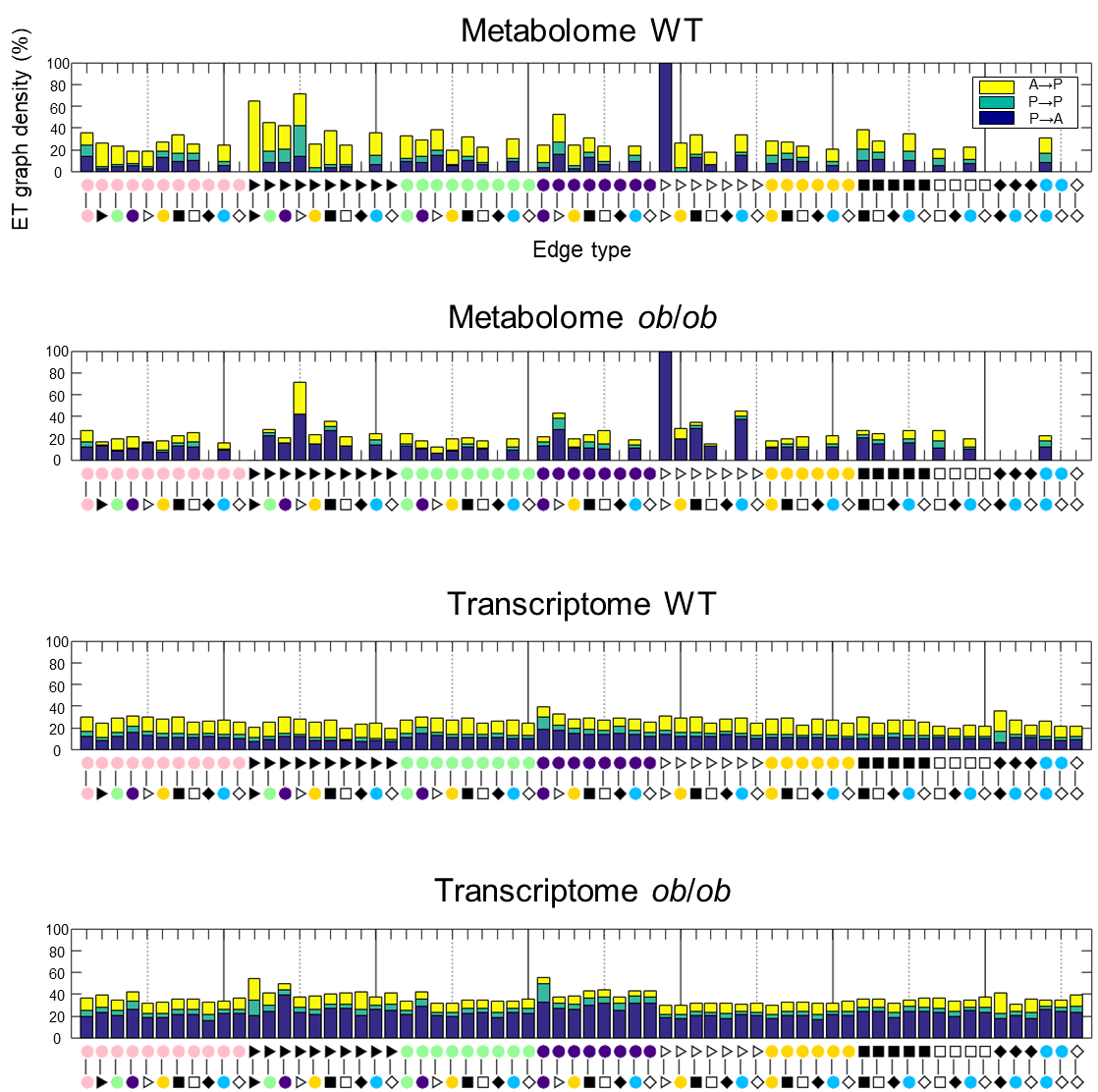


## **Fig. S3 The ratio of OGTT response edge in all 66 edge types.**

The ET graph density of each OGTT response edge (A to P, P to P, P to A) in each edge type in the metabolome and transcriptome of WT and *ob*/*ob*. Based on the definition of KEGG, metabolites and genes were annotated into 11 types, and 66 edge types were defined and the ET graph density of OGTT response edges in each edge type was examined. The node types in the horizontal axis consist of the nodes described in Figure 5 and additional node types, which are represented as follows: Biosynthesis of other secondary metabolites (closed triangle), glycan biosynthesis and metabolism (opened triangle), metabolism of cofactors and vitamins (closed square), metabolism of other amino acids (opened square), metabolism of terpenoids and polyketides (closed diamond), and xenobiotics biodegradation and metabolism (opened diamond).


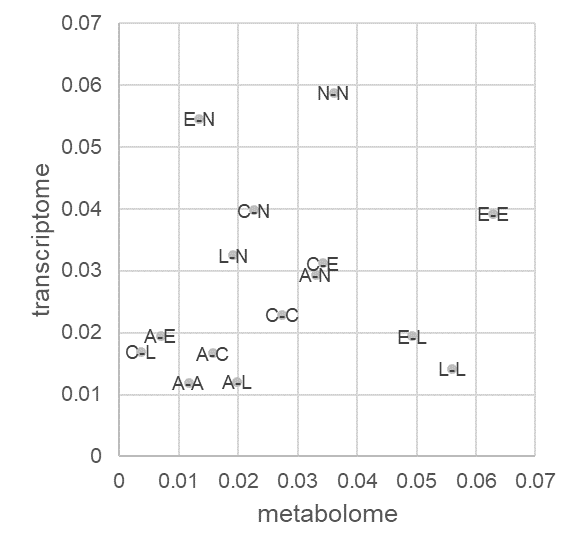


## **Fig. S4 The scatter plot of the Chi-squared histogram distance between WT and *ob*/*ob* for the metabolome and transcriptome.**

The scatter plot of the Chi-squared histogram distance of distributions of the ET graph density for OGTT response edges between WT and *ob*/*ob* for the metabolome and transcriptome in focused 15 ETs. Each ET is indicated by coupled annotations of pairwise nodes corresponding to the ET. The coupled annotations of pairwise nodes are denoted as each node annotation’s initials separated by a hyphen, respectively (A: amino acid metabolism, C: carbohydrate metabolism, E: energy metabolism, L: lipid metabolism, N: nucleotide metabolism).


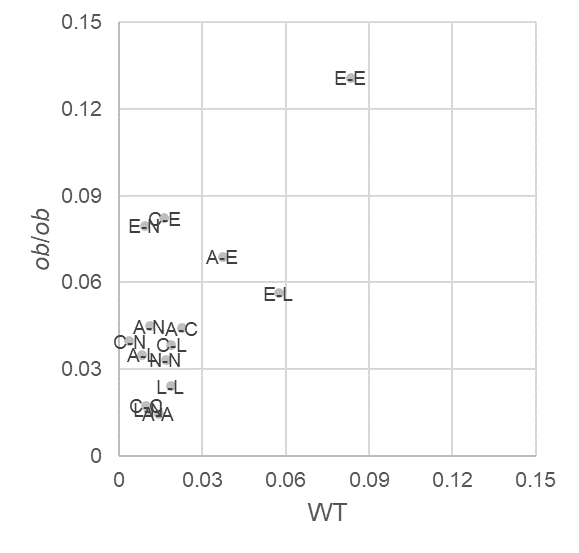


## **Fig. S5 The scatter plot of the Chi squared histogram distance between the metabolome and transcriptome for WT and *ob*/*ob*.**

The scatter plot of the Chi-squared histogram distance of distribution of the ET graph density for OGTT response edges between the metabolome and transcriptome for WT and *ob*/*ob* in focused 15 ETs. Each ET is indicated by coupled annotations of pairwise nodes corresponding to the ET. The coupled annotations of pairwise nodes are denoted as each node annotation’s initials separated by a hyphen, respectively (A: amino acid metabolism, C: carbohydrate metabolism, E: energy metabolism, L: lipid metabolism, N: nucleotide metabolism).

| Metabolome | | | | |  | Transcriptome | | | | |
| --- | --- | --- | --- | --- | --- | --- | --- | --- | --- | --- |
|  | WT (0) | WT (4) | *ob*/*ob* (0) | *ob*/*ob* (4) |  |  | WT (0) | WT (4) | *ob*/*ob* (0) | *ob*/*ob* (4) |
| WT (0) |  | 3.437E-05 | 2.678E+00 | 4.735E-02 |  | WT (0) |  | <1.0E-16 | <1.0E-16 | <1.0E-16 |
| WT (4) |  |  | 3.119E-02 | 9.695E-11 |  | WT (4) |  |  | <1.0E-16 | <1.0E-16 |
| *ob*/*ob* (0) |  |  |  | 1.096E-03 |  | *ob*/*ob* (0) |  |  |  | <1.0E-16 |
| *ob*/*ob* (4) |  |  |  |  |  | *ob*/*ob* (4) |  |  |  |  |

## **Table S1. Bonferroni corrected *p*-values of Kolmogorov–Smirnov test.**

The left and right column represent the metabolome and transcriptome, respectively. In the table, WT (0), WT (4), *ob*/*ob* (0), and *ob*/*ob* (4) stands for WT OGTT (0), WT OGTT (4), *ob*/*ob* OGTT (0), and *ob*/*ob* OGTT (4), respectively.

| "A to P" - "P to A" | | | | |
| --- | --- | --- | --- | --- |
| Ranking | ET NO. | Node1 | Node2 | Ratio (%) |
| 1 | 11 | E | L | 15.21% |
| 2 | 10 | E | E | 12.52% |
| 3 | 2 | A | C | 11.77% |
| 4 | 6 | C | C | 10.55% |
| 5 | 5 | A | N | 10.11% |
| 6 | 9 | C | N | 8.29% |
| 7 | 8 | C | L | 7.72% |
| 8 | 7 | C | E | 6.82% |
| 9 | 15 | N | N | 6.42% |
| 10 | 3 | A | E | 5.85% |
| 11 | 13 | L | L | 5.84% |
| 12 | 14 | L | N | 5.44% |
| 13 | 12 | E | N | -0.79% |
| 14 | 1 | A | A | -3.23% |
| 15 | 4 | A | L | -4.39% |

## **Table S2. The subtraction ranking of P to A from A to P in the WT metabolome.**

The ranking and the value obtained by subtracting P to A from A to P in the WT metabolome.

| "A to P" - "P to A" | | | | |
| --- | --- | --- | --- | --- |
| Ranking | ET NO. | Node1 | Node2 | Ratio (%) |
| 1 | 8 | C | L | 2.86% |
| 2 | 2 | A | C | 0.98% |
| 3 | 4 | A | L | 0.70% |
| 4 | 3 | A | E | -0.13% |
| 5 | 9 | C | N | -1.57% |
| 6 | 1 | A | A | -2.30% |
| 7 | 11 | E | L | -3.27% |
| 8 | 5 | A | N | -3.28% |
| 9 | 7 | C | E | -3.82% |
| 10 | 6 | C | C | -4.03% |
| 11 | 14 | L | N | -5.05% |
| 12 | 13 | L | L | -6.07% |
| 13 | 12 | E | N | -6.75% |
| 14 | 15 | N | N | -7.92% |
| 15 | 10 | E | E | -9.03% |

## **Table S3. The subtraction ranking of P to A from A to P in the *ob*/*ob* metabolome.**

The ranking and the value obtained by subtracting P to A from A to P in the *ob*/*ob* metabolome.

| "A to P" - "P to A" | | | | |
| --- | --- | --- | --- | --- |
| Ranking | ET NO. | Node1 | Node2 | Ratio (%) |
| 1 | 15 | N | N | 4.36% |
| 2 | 14 | L | N | 3.70% |
| 3 | 9 | C | N | 2.99% |
| 4 | 13 | L | L | 2.97% |
| 5 | 5 | A | N | 2.33% |
| 6 | 8 | C | L | 2.02% |
| 7 | 4 | A | L | 1.82% |
| 8 | 1 | A | A | 1.63% |
| 9 | 6 | C | C | 1.40% |
| 10 | 2 | A | C | 1.22% |
| 11 | 12 | E | N | -4.05% |
| 12 | 7 | C | E | -5.75% |
| 13 | 3 | A | E | -5.95% |
| 14 | 11 | E | L | -6.19% |
| 15 | 10 | E | E | -9.70% |

## **Table S4. The subtraction ranking of P to A from A to P in the WT transcriptome.**

The ranking and the value obtained by subtracting P to A from A to P in the WT transcriptome.

| "A to P" - "P to A" | | | | |
| --- | --- | --- | --- | --- |
| Ranking | ET NO. | Node1 | Node2 | Ratio (%) |
| 1 | 1 | A | A | -7.79% |
| 2 | 4 | A | L | -8.72% |
| 3 | 13 | L | L | -9.01% |
| 4 | 8 | C | L | -10.71% |
| 5 | 2 | A | C | -11.30% |
| 6 | 6 | C | C | -13.56% |
| 7 | 14 | L | N | -14.46% |
| 8 | 5 | A | N | -14.92% |
| 9 | 9 | C | N | -17.16% |
| 10 | 3 | A | E | -17.82% |
| 11 | 11 | E | L | -18.77% |
| 12 | 15 | N | N | -20.46% |
| 13 | 7 | C | E | -22.71% |
| 14 | 12 | E | N | -26.50% |
| 15 | 10 | E | E | -27.02% |

## **Table S5. The subtraction ranking of P to A from A to P in the *ob*/*ob* transcriptome.**

The ranking and the value obtained by subtracting P to A from A to P in the *ob*/*ob* transcriptome.

| increase | | | decrease | | |
| --- | --- | --- | --- | --- | --- |
|  | metabolome | transcriptome |  | metabolome | transcriptome |
| WT | 2, 3, 5, 6, 7, 8, 9, 10, 11, 13, 14, 15 | 1, 2, 4, 5, 6, 8, 9, 13, 14, 15 | WT | 1, 4, 12 | 3, 7, 10, 11, 12 |
| *ob*/*ob* | 2, 4, 8 | nothing | *ob*/*ob* | 1, 3, 5, 6, 7, 9, 10, 11, 12, 13, 14, 15 | all |

## **Table S6. The increased and decreased ET values.**

The increase or decrease of number of edges on ET in OGTT (4) is shown. The gray cells in the table indicate that the responses of each ET were same with the overall edges with respect to increase or decrease of number of edges in OGTT (4).

| metabolome | | | | |  | transcriptome | | | | |
| --- | --- | --- | --- | --- | --- | --- | --- | --- | --- | --- |
| Ranking | ET NO. | Node1 | Node2 | Dist. |  | Ranking | ET NO. | Node1 | Node2 | Dist. |
| 1 | 10 | E | E | 0.0629 |  | 1 | 15 | N | N | 0.0587 |
| 2 | 13 | L | L | 0.0560 |  | 2 | 12 | E | N | 0.0546 |
| 3 | 11 | E | L | 0.0493 |  | 3 | 9 | C | N | 0.0398 |
| 4 | 15 | N | N | 0.0361 |  | 4 | 10 | E | E | 0.0392 |
| 5 | 7 | C | E | 0.0342 |  | 5 | 14 | L | N | 0.0325 |
| 6 | 5 | A | N | 0.0330 |  | 6 | 7 | C | E | 0.0312 |
| 7 | 6 | C | C | 0.0274 |  | 7 | 5 | A | N | 0.0293 |
| 8 | 9 | C | N | 0.0226 |  | 8 | 6 | C | C | 0.0229 |
| 9 | 4 | A | L | 0.0199 |  | 9 | 3 | A | E | 0.0195 |
| 10 | 14 | L | N | 0.0192 |  | 10 | 11 | E | L | 0.0194 |
| 11 | 2 | A | C | 0.0158 |  | 11 | 8 | C | L | 0.0168 |
| 12 | 12 | E | N | 0.0135 |  | 12 | 2 | A | C | 0.0166 |
| 13 | 1 | A | A | 0.0118 |  | 13 | 13 | L | L | 0.0142 |
| 14 | 3 | A | E | 0.0071 |  | 14 | 4 | A | L | 0.0120 |
| 15 | 8 | C | L | 0.0037 |  | 15 | 1 | A | A | 0.0119 |

## **Table S7. The ranking of each ET for Chi Squared histogram distance between WT and *ob*/*ob*.**

The ranking of the ETs when ranked by descending order for the value of Chi-Squared histogram distance between WT and *ob*/*ob* calculated in Fig. 5B. Dist. stands for the Chi-Squared histogram distance.

| WT | | | | |  | *ob*/*ob* | | | | |
| --- | --- | --- | --- | --- | --- | --- | --- | --- | --- | --- |
| Ranking | ET NO. | Node1 | Node2 | Dist. |  | Ranking | ET NO. | Node1 | Node2 | Dist. |
| 1 | 10 | E | E | 0.0834 |  | 1 | 10 | E | E | 0.1307 |
| 2 | 11 | E | L | 0.0575 |  | 2 | 7 | C | E | 0.0822 |
| 3 | 3 | A | E | 0.0373 |  | 3 | 12 | E | N | 0.0796 |
| 4 | 2 | A | C | 0.0225 |  | 4 | 3 | A | E | 0.0689 |
| 5 | 8 | C | L | 0.0188 |  | 5 | 11 | E | L | 0.0562 |
| 6 | 13 | L | L | 0.0185 |  | 6 | 5 | A | N | 0.0448 |
| 7 | 15 | N | N | 0.0167 |  | 7 | 2 | A | C | 0.0441 |
| 8 | 7 | C | E | 0.0163 |  | 8 | 9 | C | N | 0.0396 |
| 9 | 1 | A | A | 0.0143 |  | 9 | 8 | C | L | 0.0385 |
| 10 | 5 | A | N | 0.0111 |  | 10 | 4 | A | L | 0.0348 |
| 11 | 14 | L | N | 0.0107 |  | 11 | 15 | N | N | 0.0331 |
| 12 | 6 | C | C | 0.0097 |  | 12 | 13 | L | L | 0.0240 |
| 13 | 12 | E | N | 0.0091 |  | 13 | 6 | C | C | 0.0173 |
| 14 | 4 | A | L | 0.0083 |  | 14 | 14 | L | N | 0.0158 |
| 15 | 9 | C | N | 0.0036 |  | 15 | 1 | A | A | 0.0145 |

## **Table S8. The ranking of each ET for Chi Squared histogram distance between the metabolome and transcriptome.**

The ranking of the ETs when ranked by descending order for the value of Chi Squared histogram distance between the metabolome and transcriptome calculated in Fig. 5C. Dist. stands for the Chi Squared histogram distance.
